# Saunders’s Medical Hand-Atlases

**Published:** 1902-03

**Authors:** 


### BOOK REVIEWS.

SAUNDERS'S MEDICAL HAND-ATLASES. Atlas and Epitomé of Bacteriology. A Text-Book of Special Bacteriologic Diagnosis. By PROFESSOR DR. K. B. LEHMANN, Director of the Hygienic Institute in Würzburg; and R. O. NEUMANN, Dr. Phil. and Med., Assistant in the Hygienic Institute in Würzburg. From the second enlarged and revised German edition. Edited by George H. Weaver, M. D, Assistant Professor of Pathology, Rush Medical College, Chicago. In two volumes. Part I., consisting of 632 colored figures on 69 lithographic plates. Part II., consisting of 511 pages of text, illustrated. Philadelphia and London: W. B. Saunders & Co. 1901. [Price, cloth, $5.00 net.]

In addition to being a most excellent work on bacteriology this book, in the very opening words of its preface, has the distinction of presenting a novelty in the shape of a glimpse behind the family curtain—something which we have never before been granted in a medical book. It is quite delightful, too, for it teaches a lesson or two; that virtue hath its own reward and that the old maxim, "If at first you don't succeed, try, try again," is a pretty good thing to tie up to as a general proposition. Here is the way the introduction begins: "For years my brother, J. F. Lehmann, the publisher in Munich, has requested me to furnish him for his medical atlases one which would simplify bacteriologic diagnosis. After I had long refused to undertake the vast labor which this would necessitate, a fortunate circumstance in the summer of 1894 led me to accept the plan."

Now imagine a reviewer with an imagination picking up this book with the best intentions in the world and running across that at the very beginning, and then wonder that the reviewing lagged. Who could help seeing in his mind's eye the soul-harrowing and pathetic picture of sordid Trade on its bended knee before the majesty of Science pleading with tearful eye and imploring voice for a book on bugs? And then the years of weary waiting on the part of brother Lehmann; the patience—it is noble; it is Joblike. But the reward came and he got the book thanks to that fortunate circumstance in the summer of 1894, which was the discovery of Dr. Neumann's talent for drawing.

With brother Lehmann we—and in "we" is included the eminent Mr. Saunders of Philadelphia, who has the distinction of having placed his name on the American edition—we should bless the summer of 1894, for it has been productive of a most remarkable book on bacteriology. It is remarkable in more ways than one. Not alone is it intelligible and hence interesting aside from its real value as a scientific work, but the illustrations in Vol. I. are probably the most perfectly pictured reproductions of bacterial life which have ever been printed. They are works of art; they are true in color and relationship and are much superior to the usual photographic reproductions and give a better general idea of the subject. This is especially true as regards the stab, streak and potato culture plates, while in the slightly magnified plate colonies the drawing gives a remarkably clear idea of the depth of the object; an end which is not always obtained when photography is wholly relied upon.

The field for photography in bacteriologic work is practically limited to the representation of individual bacteria magnified 1,000 times; and here it is unquestionably the only practical method of illustration. And a point which should not be overlooked in weighing the value of this work is that 96 per cent. of the illustrations are original, which at once removes it from any suspicion of belonging to that class of scientific publications which has been referred to in these pages heretofore on more than one occasion, as a product of a literary scrap heap. It is all fresh and it has the vigor of an honest book builded with brains.

The second or text volume is divided into two parts in which are considered general and special bacteriology. The former presents a condensed survey of the principal properties of bacteria and their aid in diagnosis; and there is a list of rules for media and stains. In the special part there is a complete description of the important varieties; a few new species have been introduced and where identical varieties have been described under various names, they have been grouped and the result is that all through the work there is noticeable an effort on the part of the author to build up a natural system. It is fortunate that brother Lehmann had such perfect patience; it is well that the summer of 1894 gave birth to such a momentous discovery. The combination has been of untold benefit to literature—and art. N.W.W.

